# On the Physical Origins of Reduced Ionic Conductivity in Nanoconfined Electrolytes

**DOI:** 10.1021/acsnano.4c18956

**Published:** 2025-03-25

**Authors:** Kara D. Fong, Clare P. Grey, Angelos Michaelides

**Affiliations:** Yusuf Hamied Department of Chemistry, University of Cambridge, Cambridge CB2 1EW, U.K.

**Keywords:** ion transport, machine learning potentials, nanoconfinement, two-dimensional materials, electrolytes, molecular simulations

## Abstract

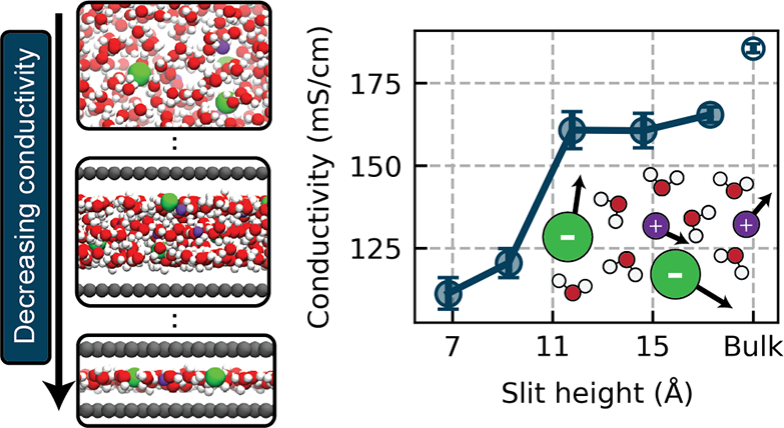

Ion transport through nanoscale pores is at the heart of numerous energy storage and separation technologies. Despite significant efforts to uncover the complex interplay of ion–ion, ion–water, and ion–pore interactions that give rise to these transport processes, the atomistic mechanisms of ion motion in confined electrolytes remain poorly understood. In this work, we use machine learning-based molecular dynamics simulations to characterize ion transport with first-principles-level accuracy in aqueous NaCl confined to graphene slit pores. We find that ionic conductivity decreases as the degree of confinement increases, a trend governed by changes in both ion self-diffusion and dynamic ion–ion correlations. We show that the self-diffusion coefficients of our confined ions are strongly influenced by the overall electrolyte density, which changes nonmonotonically with slit height based on the layering of water molecules within the pore. We further observe a shift in the ions’ diffusion mechanism toward more vehicular motion as the degree of confinement increases. Despite the ubiquity of ideal solution (Nernst–Einstein) assumptions in the field, we find that nonideal contributions to transport become more pronounced under confinement. This increase in nonideal ion correlations arises not simply from an increase in the fraction of associated ions, as is commonly assumed, but from an increase in ion pair lifetimes. By building a mechanistic understanding of confined electrolyte transport, this work provides insights that could guide the design of nanoporous materials optimized for efficient and selective ion transport.

The transport of ions through nanoscale pores and channels plays a crucial role in a diverse range of systems, spanning sustainable technologies such as water desalination^1−6^ and energy storage^7,8^ to fundamental processes in biological transmembrane transport.^9−11^ In recent years, efforts to understand the complex transport phenomena observed in these channels have been accelerated by significant advances in fabrication methods, which can now reliably generate pristine, atomically smooth nanochannels with angstrom-scale precision.^12−16^ Even in these pristine slits, however, our understanding of ion transport remains underdeveloped. Transport predictions based on continuum models break down in these highly confined systems, yielding surprising (and in many cases unexplained) behavior.^17−21^ Fully rationalizing the transport properties of confined electrolytes will inevitably require a molecular-level understanding that resolves the discreteness of individual ions and water molecules.

Molecular dynamics simulations are well-suited to accessing the time and length scales relevant to ion transport and have thus been instrumental in developing our current understanding of confined electrolyte transport. Past work has shed light on the role of specific ion effects,^22−25^ surface charge/potential,^26,27^ and, more recently, out-of-equilibrium transport phenomena beyond the linear response regime.^28−30^ However, the predictions of these types of simulations can vary significantly between studies, even in systems with nominally very similar chemistry. Consider, for example, the case of aqueous NaCl in graphene slit pores. Some studies on this system have reported that confined ion transport is slower than in bulk electrolytes,^23,24^ in line with the predictions of hindered transport theory.^31^ Others, however, observed the opposite trend: that NaCl diffusion is enhanced under confinement. Kong et al.^27^ reported approximately 2.3 times faster diffusion in a 16 Å slit relative to a bulk electrolyte, attributing this trend to the solvophobic nature of the graphene surfaces. Further work has observed more complex, nonmonotonic trends in transport, in which the conductivity and ion diffusion coefficients can either be greater or less than that of the bulk electrolyte depending on the degree of confinement.^32^ It was suggested in this work that the ordered layering of water in confinement enhances ion transport, but that this effect can be counteracted in some slit heights by changes to ion solvation and pairing.

The lack of agreement across the current body of literature suggests that ion transport is highly sensitive to the simulation setup and model parameters. Notably, the aforementioned molecular dynamics studies were performed exclusively using classical force fields, which may not be sufficiently accurate for modeling confined electrolytes. While these force fields are generally suitable for reproducing the properties of bulk electrolytes, they are not parametrized to capture the highly distorted ion solvation environments present under confinement. The accuracy of force field-based simulations is likely also limited by their relatively simplistic descriptions of electrolyte/pore interactions and the pore wall itself, as it has been demonstrated recently using both experiment^33^ and simulation^34,35^ that the properties of a confined fluid are strongly influenced by the electronic structure of the confining material. Furthermore, most force fields used to date for modeling confined electrolytes ignore electronic polarization effects, which have been shown to be crucial for realistically modeling electrolyte structure and transport in carbon nanotubes and at graphene interfaces.^36−40^

Achieving consensus on the nature of confined ion transport will undoubtedly require simulation models that more realistically capture the complex balance of ion–water, ion–pore, and water–pore interactions inherent to these systems. While a limited number of studies^37,41−43^ have reached higher levels of accuracy using ab initio molecular dynamics (AIMD), these works have exclusively characterized the structural properties of confined electrolytes, as characterizing ion transport requires time and length scales far beyond what is computationally feasible using AIMD.

Herein, we investigate ion transport in a set of prototypical nanoconfined electrolytes with first-principles-level accuracy using a neural network potential developed and validated in our previous work.^39^ Machine learning-based interatomic potentials^44−47^ such as this offer a means of retaining the high accuracy of AIMD but at orders of magnitude lower cost, allowing us to perform the long-time, large-scale simulations necessary for characterizing transport. We quantify how the electrolyte conductivity varies as a function of the degree of confinement, then establish how this trend arises based on changes in electrolyte structure, ion–ion correlations, and ion diffusion mechanisms. In doing so, we uncover insights that challenge some of the standard assumptions typically applied to confined ion transport, namely: (i) the Nernst–Einstein relation does not hold in highly confined electrolytes, despite its ubiquity across simulation, theory, and experiment, (ii) the nonideal contributions to transport are correlated not with the fraction of associated ions in solution (as is commonly assumed) but with the lifetime of ion pairs, and (iii) ions adsorbed to the graphene are found to have very similar diffusion coefficients to ions in the center of the slit, rather than the low mobility that is often assumed. In addition to these insights, which will have immediate relevance for analyzing and interpreting confined ion transport behavior, we envision that the mechanistic understanding developed in this work will ultimately contribute toward the development of nanoporous materials with high conductivity and ion selectivity for applications ranging from energy storage to filtration.

## Results and Discussion

### Ionic Conductivity Decreases under Nanoconfinement

**Figure 1:**
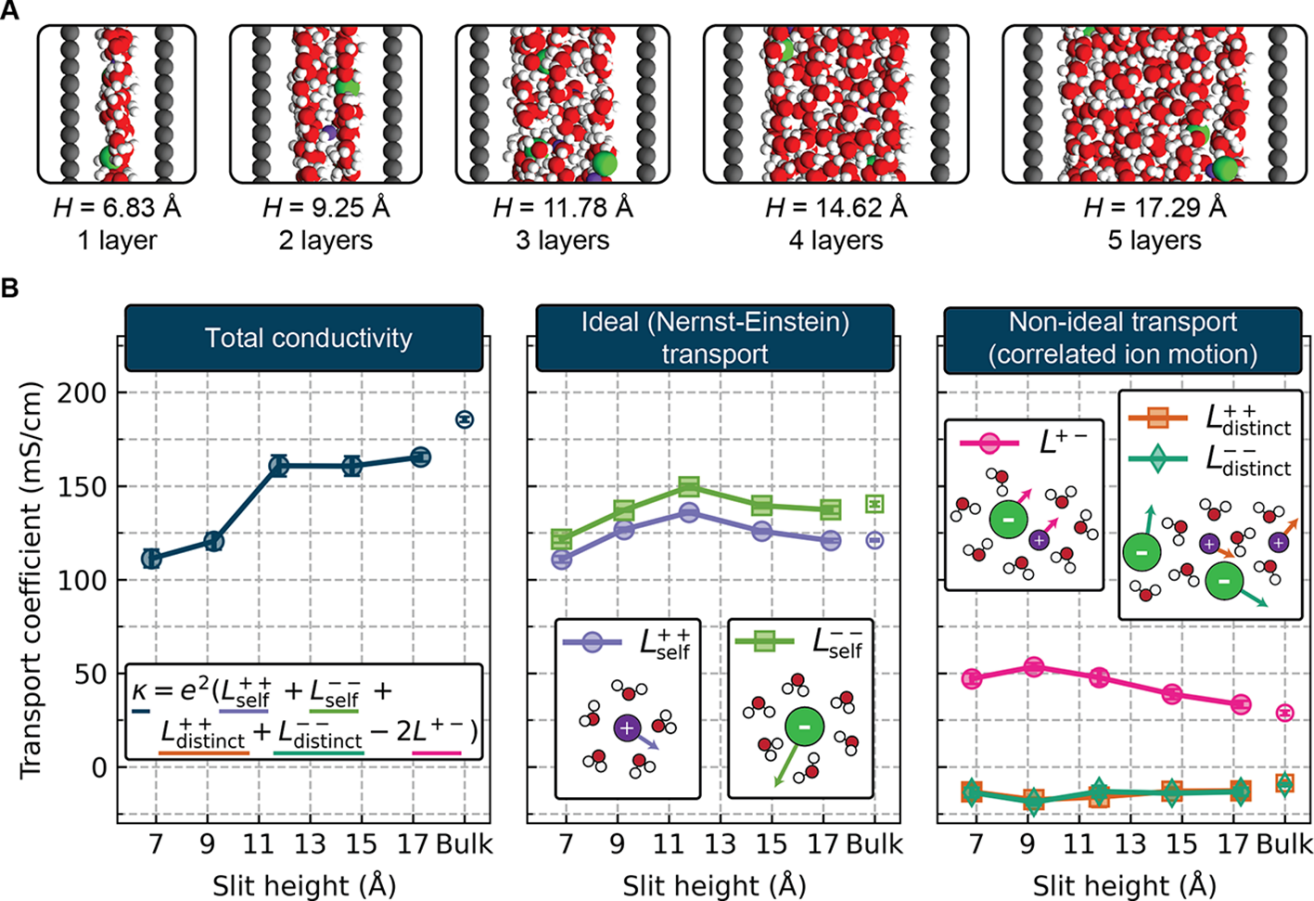
(A) Snapshots of each of the nanoconfined electrolytes studied herein. Only a small portion of the full simulation cell is shown; full cells are given in Figure S1. (B) Ion transport parallel to the graphene sheets as a function of slit height, *H*. The left panel gives the total ionic conductivity κ. The middle panel shows the two Nernst–Einstein transport coefficients,  and , and the right panel shows the nonideal transport coefficients, , , and *L*^+–^. In each panel, the rightmost data point corresponds to the bulk electrolyte. Note that while the legend denotes each *L*^*ij*^, the plotted data corresponds to *e*^2^*L*^*ij*^ such that the units are mS/cm. Error bars are given as the standard deviation of five independent replicate simulations.

In this work, we investigate the in-plane transport properties of aqueous NaCl confined within rigid graphene slit pores. We consider slits containing one, two, three, four, and five layers of water, in which *H*, the distance between the graphene sheets, is 6.83, 9.25, 11.78, 14.62, and 17.29 Å. Each of these systems is depicted in Figure 1A. These slit heights are comparable to those that can be fabricated experimentally using a van der Waals assembly method, where atomically flat sheets are separated by spacers made from a precise number of graphene layers.^12^ We determine the total density of electrolyte within each slit through an equilibration procedure in which one of the graphene sheets is allowed to move under the force of a piston applying a pressure of 1 atm. The equilibrium slit height obtained from this procedure reflects the preferred layering of water and ions in the slit; see Figure S4 for the density profiles of each system. In each slit, the ratio of ion pairs to water molecules is fixed at approximately 1:55, corresponding to a 1 M solution in a bulk electrolyte. Details on the exact composition of each system are provided in the Supporting Information. All simulations were run using a neural network potential developed in our previous work.^39^ The model was trained on reference calculations at the revPBE-D3 level of theory,^48,49^ which has been shown to accurately capture both water-graphene^50^ and NaCl-water^51,52^ interactions. In addition to the extensive validation presented in our previous work, in Figures S2 and S3 we present further validation against the dynamics of the underlying reference method as well as experimental bulk conductivity data.

The total ionic conductivity κ as a function of slit height *H* is provided in the left panel of Figure 1B. We observe that the largest slit studied, *H* = 17.29 Å, exhibits 11% lower conductivity than the bulk electrolyte, with a further decrease in the conductivity upon confining the electrolyte to a bilayer and monolayer (*H* = 9.25 Å and *H* = 6.83 Å, respectively). The magnitude of this decrease in conductivity is comparable to what would be expected based on the limited experimental data available for systems such as this: Esfandiar et al.^13^ characterized aqueous NaCl transport in bilayer electrolytes confined to slit pores fabricated by van der Waals assembly, observing a 32% decrease in ionic conductivity relative to a bulk electrolyte. We observe a 35% decrease in our bilayer system, albeit at a higher concentration than was used by Esfandiar et al.^13^

In order to understand the factors contributing to the ionic conductivity trend, we can decompose the total conductivity κ into its constituent Onsager transport coefficients, *L*^*ij*^.^53,54^ These transport coefficients are computed using Green–Kubo relations (eqs 3 and 4 in the Methods section) and are related to the conductivity via
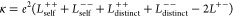
1where *e* is the elementary charge. These transport coefficients capture the various types of ion motion which collectively give rise to the overall transport behavior in the system. The quantities  and  capture the ideal or Nernst–Einstein contributions to transport arising from self-diffusion of the cations and anions, respectively. As shown in the middle panel of Figure 1B,  and  vary nonmonotonically with slit height, reaching a maximum in the slit with three layers of water. The terms , , and *L*^+–^ capture nonideal contributions to the conductivity arising from correlations in ion motion. For example, correlated motion of cations and anions (such as an ion pair moving together) will result in increased *L*^+–^ and, by eq 1, decrease the conductivity. These three nonideal transport coefficients are provided in the right panel of Figure 1B. We observe that the decrease in conductivity between the bulk electrolyte and the *H* = 17.29 Å system arises from both small decreases in  and  as well as increases in the magnitude of each of the nonideal transport coefficients. The drop in conductivity upon further confining the electrolyte is attributed to both decreases in  and  and increased *L*^+–^. Note that the anion and cation transport coefficients follow very similar trends, such that the transference number remains nearly constant across all degrees of confinement (Figure S5).

In the following sections, we aim to rationalize the trends in each of the Onsager transport coefficients to understand the physical processes contributing to decreased conductivity under confinement. We note, however, that rigorously characterizing transport in confined systems presents some technical challenges. Most notably, the Green–Kubo relations used to compute *L*^*ij*^ include a factor of 1/*V*, where *V* is the volume of the electrolyte. This volume is not uniquely defined in confined systems due to ambiguities in defining the solid/liquid interface. As in previous work,^24^ we define the volume as *V* = *L*_*x*_*L*_*y*_ (*H* – 2*r*_C_), where *r*_C_ is the van der Waals radius of carbon, 1.7 Å, *H* is the separation distance between the graphene layers, and *L*_*x*_ and *L*_*y*_ are the dimensions of the graphene sheet in the *x*- and *y*-directions, respectively. This choice of volume will influence the values of *L*^*ij*^ but should not qualitatively affect any of the trends presented in this work (see Figure S11, in which we show how each of the transport coefficients changes after a ±20% change in *r*_C_).

### Electrolyte Density Dictates Nernst–Einstein Transport

In this section, we explore the trends in  and , the Nernst–Einstein contributions to transport, reported in Figure 1B. These transport coefficients are directly related to the ion self-diffusion coefficients via
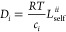
2where *RT* is the thermal energy and *c*_*i*_ is the concentration of species *i*. As with the transport coefficients reported above, we consider self-diffusion only in the plane parallel to the graphene sheets.

**Figure 2:**
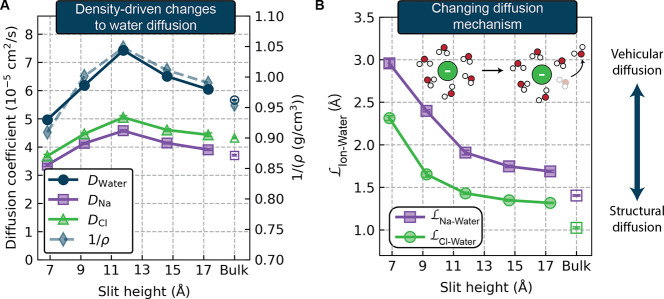
Factors affecting Nernst–Einstein transport. (A) Diffusion coefficients of each species as a function of slit height (left axis), overlaid with the inverse of the solution density ρ (right axis). (B) , the diffusion length of a water molecule in an ion’s first solvation shell, as a function of slit height. Higher values correspond to more vehicular diffusion, while lower values indicate more structural diffusion.

We find that the primary factor influencing  and  is the overall density of the electrolyte, where the latter is determined by the piston equilibration method described above. In Figure 2A, we overlay the electrolyte’s self-diffusion coefficients and the inverse of the solution density as a function of slit height. We observe a very strong correlation between these quantities, with higher densities yielding lower self-diffusion coefficients of all species. The nonmonotonic trend in water dynamics resulting from density/layering effects has been reported previously for both viscosity^55^ and diffusion coefficients^56^ based on classical force field-based simulations of pure water in graphene slit-pores, in which oscillations were observed based on whether the slit heights were commensurate or incommensurate with full, well-separated water layers. We observe that nonmonotonic water transport also translates to very similar trends in sodium and chloride ion diffusion.

To further demonstrate the importance of solution density in determining electrolyte diffusion, we have performed additional simulations for some of the slit heights in which the density is fixed at a constant value, rather than the equilibrium value obtained from the piston-based procedure. These systems will be subject to artificial pressure between the electrolyte and graphene sheets and thus may not correspond to experimentally relevant setups, but they allow us to more directly probe the role of density on electrolyte transport. As seen in Figure S17, while the overall trends in conductivity and the nonideal transport coefficients remain unchanged, keeping the electrolyte density fixed suppresses variations across slit height in the diffusion coefficients of all species. The range of sodium ion diffusion coefficients across all slit heights, for example, decreased from 1.2 × 10^–5^ cm^2^/s in Figure 2A to 1.2 × 10^–6^ cm^2^/s in the fixed density simulations, a 90% decrease. The chloride ion and water diffusion coefficient ranges similarly decreased by 79 and 62%, respectively. Further discussion of these results is provided in the SI.

The strong correlation between solution density and diffusion has important implications for the interpretation of past works studying transport in similar confined systems, in which there exists a range of approaches for choosing the packing/density of a slit pore. Some studies apply the piston-based equilibration approach used here^57−59^ or allow the confining wall to move freely (a zero pressure condition),^60^ while others aim to match the overall density in the pore to experimental bulk density values.^55,61^ Other works allow the slit to fill in contact with a bulk solution reservoir,^27,56,62^ a strategy which is only computationally feasible using force field-based simulations. More broadly, questions related to properly packing nanoconfined slits and rigorously defining the pressure in these systems comprise an active and controversial area of research.^63−65^ We hypothesize that differences in slit packing across the literature may be responsible in part for the wide discrepancy of confined ion transport trends described in the Introduction.

### Diffusion Becomes More Vehicular under Confinement

In addition to changes in ion self-diffusion induced by variations in the solution density, we also observe that the mechanism for ion diffusion changes with the degree of confinement. This mechanism can be quantified by the diffusion length  describing the characteristic distance for an ion to diffuse together with a water molecule in its first solvation shell. This diffusion length, illustrated schematically and plotted for both ions in Figure 2B, allows us to classify ion transport as either vehicular or structural.^66^ When  is large, an ion travels an appreciable distance with its first solvation shell intact, and its diffusion is classified as vehicular. In contrast, structural diffusion is characterized by lower , in which water molecules in the first solvation shell are frequently exchanged. Structural diffusion is typically associated with faster diffusion, a trend which can be loosely rationalized via the Stokes–Einstein–Sutherland (SES) equation: . Here, η is the viscosity of the fluid and *R*_H_ is the hydrodynamic radius of the particle. Under a vehicular diffusion mechanism, an ion’s hydrodynamic radius increases to effectively include the size of its first solvation shell, resulting in slower diffusion. The SES equation cannot be quantitatively applied to ion transport, especially in these confined systems, where additional factors will play a role such as changes in ion solvation structure (see Figure S12). Previous molecular dynamics simulations based on classical force fields, for example, have noted that distorted hydration shells and reduced coordination numbers at an interface are associated with slower ion diffusion.^22,43^ However, classifying transport as vehicular or structural provides a useful qualitative lens for interpreting transport trends. For example, we can rationalize the fact that the chloride exhibits a higher self-diffusion coefficient than the sodium ion based on its more structural diffusion mechanism, which is consistent with chloride’s weaker hydration energy.

We observe that the ion–water diffusion length increases as the degree of confinement increases. This shift toward a more vehicular diffusion mechanism is particularly pronounced in the two most confined systems, which is consistent with the decreases in  and  observed in these slits. Such a trend is likely due to the increased steric constraints on solvation imposed by the graphene walls, which raise the barrier for water molecules to leave the ion’s solvation shell. Indeed, in the most confined system we see higher free energy barriers for a water molecule to transition from the first to the second solvation shell of an ion (see the ion–water potentials of mean force in Figure S6). Together with the density-driven changes to diffusion, this changing diffusion mechanism gives rise to the observed Nernst–Einstein transport trends observed in Figure 1B. Notably, these trends in diffusion mechanism and diffusion coefficient do not coincide with the trend in water hydrogen bond lifetimes (Figure S13), suggesting that there exists a degree of decoupling between the translational and rotational motion of the water in these systems.

### Nonideal Contributions to Transport Become More Significant under Confinement

**Figure 3:**
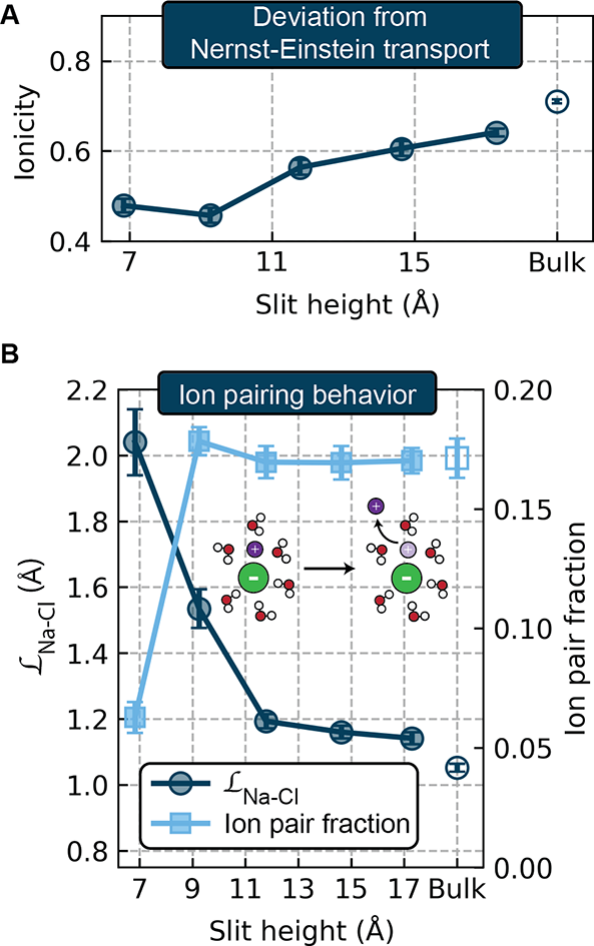
(A) Ionicity as a function of slit height. (B) Ion pairing behavior as a function of slit height. The left axis gives , the diffusion length of an ion pair, while the right axis gives the average fraction of ions existing as contact ion pairs.

In addition to changes in Nernst–Einstein transport, the role of nonideal contributions to transport arising from ion–ion correlations also changes as a function of the degree of electrolyte confinement. The collective impact of these nonideal contributions to transport can be quantified via the ionicity (also known as the inverse Haven ratio), which is defined as the ratio of the total conductivity and the Nernst–Einstein conductivity, i.e., . An electrolyte obeying ideal solution or Nernst–Einstein transport would have an ionicity value of one. As shown in Figure 3A, the bulk electrolyte exhibits an ionicity of 0.71, while the ionicity decreases in confined systems down to a value of 0.46 for the bilayer electrolyte. This change reflects the trends in the nonideal transport coefficients shown in the right panel of Figure 1B.

While the breakdown of the Nernst–Einstein relation (nonunity of the ionicity) in nanoconfined electrolytes has been recognized in recent work,^36,67^ ideal solution transport assumptions are widely used in molecular dynamics-based studies, continuum modeling, and experimental work on confined systems.^22,27,68−70^ Our results suggest that such assumptions would give rise to significant error, in this case a 52% overestimation of the conductivity in the monolayer electrolyte.

Deviations from Nernst–Einstein transport are conventionally rationalized in terms of ion pairing.^71^ A charge-neutral ion pair will diffuse, but it will not migrate under an electric field; it will thus contribute to the Nernst–Einstein conductivity but not the actual conductivity, lowering the ionicity. As such, the ionicity is frequently used as a direct measure for the degree of ion dissociation in experimental studies.^72−74^ Based on this intuition and our observed decrease in ionicity as the degree of confinement increases, we might expect more ion pairing in the most confined electrolytes. However, we observe the opposite trend: the most confined electrolyte has a lower degree of ion pairing (Figure 3B). This ion pairing trend was explored in our prior work,^39^ where we suggest that polarization of the graphene sheets weakens electrostatic interactions in solution and thus decreases the favorability of ion pairing. Such effects only become pronounced in the monolayer electrolyte, where the ions directly interact with both graphene sheets simultaneously.

The poor agreement between the trends in ionicity and the fraction of associated ions has been observed in numerous other systems, from superconcentrated electrolytes to polymer-based electrolytes.^75−78^ In fact, it is becoming increasingly recognized that a purely static analysis of ion pairing is insufficient to capture dynamic ion correlations.^54,79^ Several studies have found that a dynamic measure of ion pairing, as quantified by the average lifetime of an ion pair or the ion pair diffusion length , correlates much more strongly with the ionicity.^75,76^ The ion pair diffusion lengths for the nanoconfined electrolytes studied here are plotted in Figure 3B;  increases as the slit height decreases, suggesting that the steric constraints imposed by the graphene sheets increase the free energy barrier for an ion pair to break apart. For slits with two or more layers, we indeed observe that the increasing  trend as the slit height decreases closely parallels the trend in decreasing ionicity. Thus, while the number of ion pairs remains relatively constant across these systems, each ion pair diffuses together for a longer distance, yielding more correlated cation–anion motion (*L*^+–^) and lower ionicity. In the monolayer electrolyte, however, we hypothesize that the ion pairing fraction and  both contribute to the ionicity: the simultaneous increase in diffusion length and decrease in the fraction of ion pairs collectively give rise to a very small change in ionicity relative to the bilayer system.

### Ion Transport Varies Minimally across the Height of the Slit

**Figure 4:**
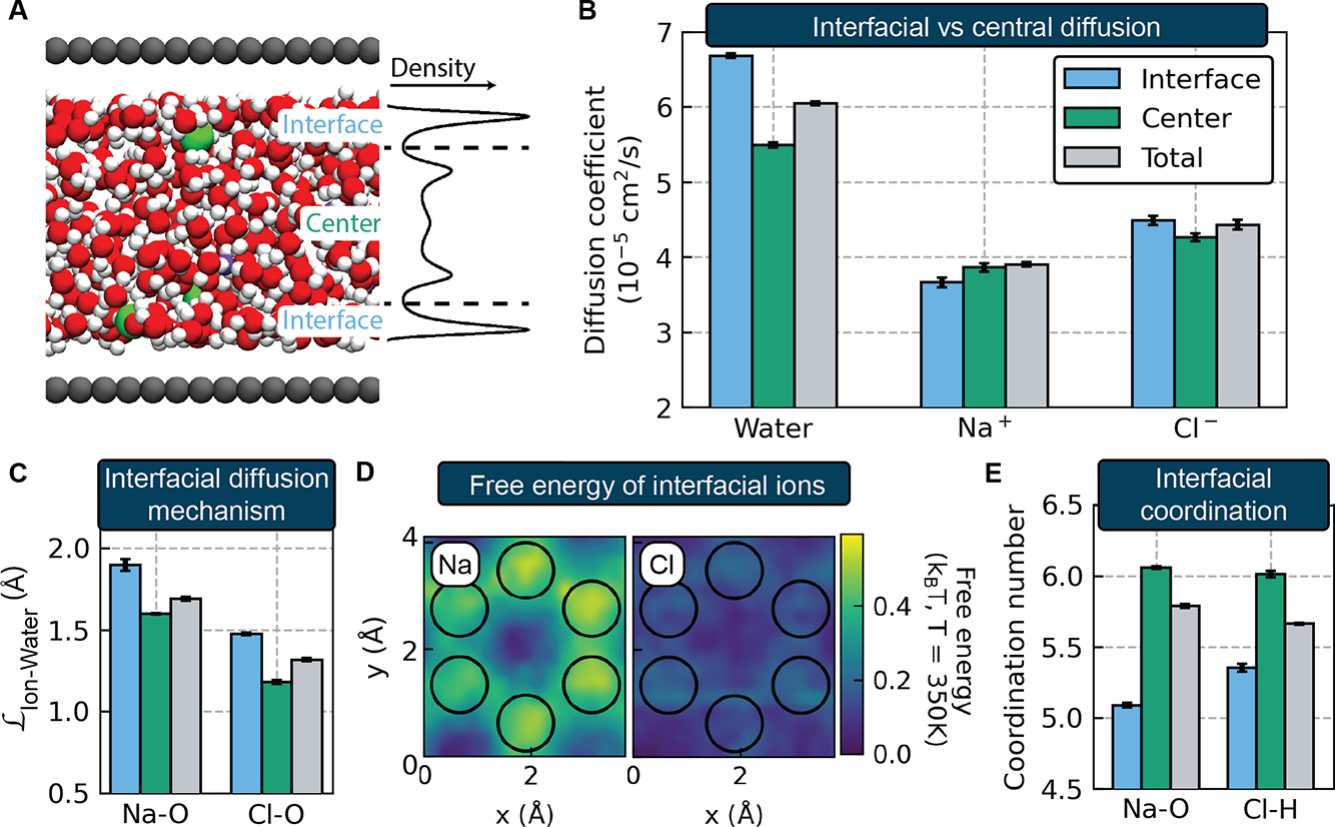
Comparing transport at the graphene interface and in the center of the slit for the *H* = 17.29 Å system. (A) Schematic illustrating how the first minimum of the density profile is used to delineate interfacial versus central species. (B) Diffusion coefficients of each species at the interface, in the center of the slit, and averaged over the entire slit (total). (C) Diffusion length of water molecules in the ions’ first solvation shell, , at the interface, in the center of the slit, and averaged over the entire slit. (D) Free energy profile of interfacial ions projected onto the graphene lattice. Black circles denote the positions of carbon atoms. (E) Ion coordination numbers at the interface, in the center of the slit, and averaged over the entire slit.

Having analyzed the overall transport behavior in each of our confined electrolyte systems, we now turn to characterizing how transport differs in the interfacial electrolyte layers compared to the center of the slit. The border between these regions is defined as the first minimum in the density profile for each species, as illustrated in Figure 4A. Figure 4B compares the self-diffusion coefficient of each species of the electrolyte in the interfacial and central portions of the slit for the largest system studied herein (5 water layers, *H* = 17.29 Å).

We observe that the water self-diffusion coefficient is 22% larger at the graphene interface than in the center of the slit, a trend that is consistent with the very low friction observed in both simulations^80−83^ and experiment^16,84^ for water/graphene interfaces. Enhanced interfacial diffusivity has been observed previously in classical force field-based simulations of water at hydrophobic interfaces^85^ and is consistent with continuum hydrodynamics-based analyses assuming perfect slip boundary conditions (infinite slip length).^85,86^

Based on the close agreement between the water and ion self-diffusion coefficient trends observed in Figure 2A, one might expect the ions to also diffuse significantly faster in the interfacial layer. However, relatively little change in the ion self-diffusion coefficients is observed: sodium ions diffuse approximately 5% slower at the interface, while interfacial chloride ions diffuse approximately 5% faster. We attribute this relative uniformity in ion diffusion coefficients across the height of the slit to a cancellation of factors. On the one hand, the interfacial ions are diffusing in a less viscous medium due to the water’s larger diffusion coefficient. This effect, however, is countered by a changing interfacial diffusion mechanism. As shown in Figure 4C, both the sodium and chloride ions exhibit more vehicular diffusion mechanisms (longer ion–water diffusion lengths) at the interface, which is associated with slower self-diffusion.

Interfacial ion diffusion is also likely slowed by interactions between the ions and the graphene surface. Figure 4D shows the free energy of interfacial sodium and chloride ions as a function of position on the graphene surface. This quantity is defined as −*k*_B_*T*ln*P*_ion_, where *P*_ion_ is the spatial probability distribution of an ion in the interfacial layer projected onto the graphene lattice.^80^ Here we observe that the sodium ion experiences a more corrugated energy landscape at the graphene surface than the chloride does. This difference in surface interaction is related to the fact that the sodium ions lose more of their first solvation shell when adsorbing to the interface than the chloride ions, as quantified in the coordination numbers presented in Figure 4E. We hypothesize that these differences in surface interactions and interfacial solvation give rise to the contrasting trends seen for the cation and ion, in which sodium ions diffuse most quickly in the center of the slit but chloride ions diffuse most quickly at the interface.

## Conclusions

In this work, we have investigated ion transport in aqueous sodium chloride confined within nanoscale graphene slit pores. Our simulations of these confined electrolytes were carried out with first-principles-level (revPBE-D3) accuracy with the help of a machine learning potential developed and validated in our previous work.^39^ We have established that the ionic conductivity in highly confined electrolytes is significantly lower than that in bulk electrolytes. By computing each of the Onsager transport coefficients, we demonstrate that this decrease in conductivity arises from changes in both ion self-diffusion (Nernst–Einstein transport) and ion–ion correlations. We find that the overall density of the solution is the major factor dictating the self-diffusion coefficients of all species in the electrolyte, and that the ions’ diffusion mechanism shifts toward more vehicular motion as the degree of confinement increases. We further observe a marked deviation from the Nernst–Einstein relation which is commonly applied to study these electrolytes. This deviation arises from an increase in the nonideal contributions to transport, particularly cation–anion correlations. While such cation–anion correlations are conventionally described in terms of a static or structural picture of ion pairing, i.e., the fraction of associated ions, we find that a dynamic picture of ion pairing based on the average diffusion length of ion pairs is necessary to rationalize this correlated ion transport. Finally, we demonstrate that while water diffuses faster at the graphene interface than in the center of the pores, ion diffusion is largely unchanged across the height of the slit. This observation challenges the standard assumption that surface-bound ions exhibit low mobility.^22,43,87^

By studying the transport behavior of a relatively simple electrolyte in idealized slit pores, this work lays the foundation for developing a comprehensive, predictive understanding of transport in more complex nanoconfined electrolytes. Future work will explore the effect of surface chemistry on confined ion transport, from the impact of the electronic structure of the pore wall to the role of defects and functional groups, as well as specific ion effects and the behavior of mixed electrolyte solutions. These future efforts will be instrumental in designing optimized nanoconfined environments that enable fast and selective ion transport for applications in energy storage and separations technologies.

## Methods

### Machine Learning Potential

In this work, we drive molecular dynamics simulations using a committee of eight Behler–Parrinello neural network potentials (NNP) developed in our previous work.^39^ While the technical details regarding the NNP architecture, training set curation, and validation are provided in our previous publication,^39^ here we summarize the key features of the model. Our NNP is trained based on density functional theory (DFT) reference data at the revPBE-D3 level of theory.^48,49^ The training set includes both bulk aqueous NaCl structures as well as confined structures across a range of concentrations and slit heights. The NNP architecture used here only captures short-range interactions within the 12 Bohr cutoff of the atom-centered symmetry functions^88^ encoding the chemical environments within the system. In order to capture long-range electrostatic effects, we include a Coulomb baseline, in which each atom is assigned a fixed partial charge (ions are ±1, carbon atoms are zero, and water molecules have the charges of the SPC/E model). These charges give rise to the Coulombic energy of a system, *E*_Coul_. We can then decompose the total energy from DFT as *E* = *E*_sr_ + *E*_Coul_, where *E*_sr_ is a short-ranged contribution. The NNP is trained only on *E*_sr_ and the corresponding short-ranged forces. When running molecular dynamics, we propagate the dynamics by summing together both the short-ranged contributions from the NNP and the Coulombic baseline. Note that the model does not capture any charge transfer between the graphene sheets and the electrolyte, for example through ion chemisorption.

In our previous study,^39^ we validated the performance of our NNP based on both energy/force errors relative to the DFT reference method as well as comparisons to an ab initio molecular dynamics (AIMD) trajectory, from which we obtained radial distribution functions, density profiles, and the vibrational density of states. Further validation of the model for this work is given in the SI, including comparison to AIMD velocity autocorrelation functions, comparison to experimental conductivity data in the bulk electrolyte, and assurance that the model uncertainty (quantified by the disagreement between the eight committee members) remains low throughout all simulations.

### Simulation Methods

Molecular dynamics simulations were performed using the LAMMPS interface of the n2p2 code.^89,90^ Simulation cells were periodic in all three directions. In order to avoid spurious interactions between periodic images of the slab in the *z*-direction (perpendicular to the graphene sheets), we included a layer of vacuum at least three times the slit height between periodic images of the slab and applied the Yeh and Berkowitz correction.^91^ Coulombic interactions were evaluated with the PPPM method. Equations of motion were integrated using the velocity-Verlet algorithm with a time step of 0.5 fs. The interatomic forces used for this numerical integration are based on the average prediction of all eight committee members of the neural network potential. All simulations were carried out in the *NVT* ensemble using a Nosé–Hoover style thermostat with a damping parameter of 100 fs. We chose to run all simulations at a temperature of 350 K to avoid the complicating influence of phase transitions in the bilayer electrolyte, which were observed at 300 K (see the SI for more details). Simulation cells were initially prepared by randomly packing the desired number of water molecules and ions between two graphene sheets. These initial structures were subsequently equilibrated with a 1 ns simulation using a classical force field (SPC/E water model, NaCl parameters from Dang,^92^ and water–carbon parameters from Werder^93^), followed by a 1 ns simulation using the NNP. Production runs were then carried out for 5 ns. For each system, we performed five independent replicate simulations. All error bars in the text reflect the standard deviation among these replicates.

The NaCl-to-water ratio in each system was fixed at approximately 1:55, which corresponds to a 1 M solution in a bulk electrolyte. Note this ion-to-water ratio may not correspond to that which would be observed if the slit pore was in contact with a bulk 1 M electrolyte reservoir.^94,95^ However, given that it remains challenging to quantify the proper ion-pore concentration through both simulation and experiment,^22,96^ choosing to fix the ion-to-water ratio enables a clean comparison across all slit heights. Each graphene sheet has dimensions of approximately 45 Å by 45 Å, with the precise value adjusted to accommodate the target ion-to-water ratio as closely as possible. The equilibrium height of the slit and thus the density of the solution was determined via additional simulations in which one of the graphene sheets was allowed to move in the *z*-direction (perpendicular to the sheet) under the force of a piston applying 1 atm of pressure. Using this method, we prepared five slits that contained 1–5 layers of water; see the density profiles in Figure S4. Table S1 provides the precise dimensions and number of molecules in each system. Note that while the graphene is allowed to move vertically during these piston simulations, we hold the position of all carbons fixed during our production simulations.

### Data Analysis

Onsager transport coefficients were computed using the following Green–Kubo relation:^53^
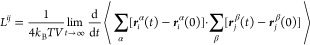
3Here, *k*_B_*T* is the thermal energy and ***r***_*i*_^α^(*t*) is the position vector of particle α relative to the center-of-mass position of the entire electrolyte (excluding the carbon atoms). As we only consider transport in the *xy*-plane (parallel to the graphene sheets), ***r***_*i*_^α^(*t*) is a two-component vector, and we include a factor of 1/4 in the prefactor of eq 3 rather than the factor of 1/6 used in isotropic systems. The angular brackets denote averaging over time origins within the trajectory, and the indices *i* and *j* denote the species type (cation or anion). As mentioned above, we define the volume *V* of the electrolyte as *V* = *L*_*x*_*L*_*y*_ (*H* – 2*r*_C_), where *r*_C_ is the van der Waals radius of carbon, 1.7 Å, *H* is the separation distance between the graphene layers, and *L*_*x*_ and *L*_*y*_ are the dimensions of the graphene sheet in the *x*- and *y*-directions, respectively. The same definition of volume is used when computing the density in Figure 2A. The self-transport coefficients are computed analogously via
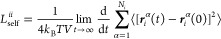
4and the distinct terms were computed by . The total conductivity is then obtained from eq 1. Diffusion coefficients are computed via
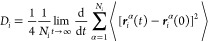
5Note that combining eqs 4 and 5 yields eq 2. Finite-size corrections are applied to all of the transport coefficients described above, as discussed in the SI.

In the diffusive regime, the quantity in angular brackets in eqs 3, 4, or 5 must be linear with respect to time, e.g.,  and , with β = 1. In Table S2, we report β for each of the transport coefficients to demonstrate that our simulations are long enough to reach the linear regime. We additionally compare the transport coefficients computed from a run twice as long as our standard run time (5 ns) and find agreement between the two sets of data (Figure S8), further demonstrating the convergence of our results. Representative data for the quantity in angular brackets for each of the transport coefficients is given in Figure S7. The standard procedure for fitting eqs 3, 4, or 5 to obtain the corresponding transport coefficient entails choosing a time regime in which the quantity in angular brackets is approximately linear, then performing a linear regression on the quantity in angular brackets to obtain a slope. However, the ordinary method of least-squares is only an optimal fitting procedure if each of the data points has the same finite variance. The quantities in angular brackets in eqs 3, 4, or 5, however, exhibit heteroscedasticity: because we are averaging over all time origins within the trajectory, data points at longer times have higher variance. In this case, we can use weighted least-squares to fit the data, where the weights are the reciprocal of the variance of the quantity in angular brackets. By providing higher weights for the short-time data (where we have better statistics), we observe consistently lower error bars in our transport coefficient measurements. All transport coefficients are fit over two decades of time, from 1 to 100 ps, with the initial equilibration period excluded from the analysis.

We distinguish between self-diffusion at the interface and in the center of the slits as follows. First, we define a cutoff distance based on the first minimum of the density profile for each species (water, sodium ions, and chloride ions); if the distance between the species and the graphene sheet is less than this cutoff distance, it is deemed an interfacial species. Next, for each ion and water molecule, we slice the full simulation trajectory whenever the species moves across the cutoff distance, thus generating short trajectories in which the species is either only at the interface or only in the center of the slit. Both the diffusion coefficients in Figure 4A and the ion–water residence times presented in Figure 4C are computed from these split trajectories. We only consider trajectory slices that are at least 5 ps long to ensure that we can adequately probe the diffusive regime. Furthermore, we omit the first ps from each sliced trajectory from the analysis to remove edge effects. The total diffusion coefficients reported in Figure 4B are equivalent to those reported for the *H* = 17.29 Å system in Figure 2A and are obtained from the full simulation trajectories.

Diffusion lengths for both ion pairs and water molecules in ions’ first solvation shells are computed via , where τ is the average lifetime of the pair of species. This lifetime is evaluated using the correlation function *P*_αβ_(*t*) = ⟨*H*_αβ_(*t*)*H*_αβ_(0)⟩, where *H*_αβ_(*t*) is one if particles α and β are neighbors at time *t* and zero otherwise.^66,97,98^ Two particles are defined as neighbors if they are within a specified cutoff distance, which we take to be the first minimum of the radial distribution function. The residence time is defined as the time taken for *P*_αβ_(*t*)/*P*_αβ_(0) to decay to a value of 1/*e*. The correlation functions for τ_Na–Cl_, τ_Na–O_, and τ_Cl–O_ are provided for each slit height in Figure S9, and the values for τ_Na–Cl_, τ_Na–O_, and τ_Cl–O_ are given in Figure S10. The diffusion coefficient *D* is taken to be the average of the two species’ diffusion coefficients. For example, in computing , *D* is 0.5(*D*_Na_ + *D*_Cl_). As the diffusion coefficient trends for all species align very closely, this choice of *D* does not affect the trends in  versus slit height.

## Data Availability

All data required to reproduce the findings of this study is available at https://github.com/water-ice-group/confined-ion-transport.

## Supporting Information Available

Additional details on system setup and composition; validation of the machine learning potential; additional system characterization (density profiles, potentials of mean force, etc.); discussion of finite size effects; simulations at fixed electrolyte density; transport behavior at 300 K; and additional Supporting Information available online at https://github.com/water-ice-group/confined-ion-transport (PDF)

## Notes

A preprint version of this article was deposited on the ChemRxiv repository.^99^
