# Supplementary material for: On the Physical Origins of Reduced Ionic Conductivity in Nanoconfined Electrolytes
Source: ACS Nano. 2025 Mar 25;19(13):13191–201. doi: 10.1021/acsnano.4c18956 (PMC11984311; doi:10.1021/acsnano.4c18956)
Supplement: Supplementary file 1 — nn4c18956_si_001.pdf [file nn4c18956_si_001.pdf]

Supporting Information: On the physical origins of reduced ionic conductivity in nanoconfined electrolytes

Kara D. Fong,* Clare P. Grey,* and Angelos Michaelides*

Yusuf Hamied Department of Chemistry, University of Cambridge, Cambridge, Lensfield Road, CB2 1EW

E-mail: kdf22@cam.ac.uk; cpg27@cam.ac.uk; am452@cam.ac.uk

Contents

1 Additional simulation details **S-2**
1.1 System composition S-2
1.2 Machine learning potential validation S-2

2 Additional system characterization **S-5**

3 Finite size effects **S-11**

4 Fixed density simulations **S-14**

5 Transport at 300K **S-15**

References **S-16**

1 Additional simulation details

1.1 System composition

Table S1: Slit dimensions and number of water molecules and ion pairs for each system studied. The size of the graphene sheet varies slightly across each system to accommodate the target ion-to-water ratio as closely as possible.

Slit Height (Å)	Graphene dimensions (Å x Å)	Number of waters	Number of NaCl
6.83	49.40 x 47.06	277	5
9.25	44.46 x 47.06	386	7
11.78	46.93 x 47.06	558	10
14.62	44.46 x 47.06	734	13
17.29	44.46 x 47.06	926	17

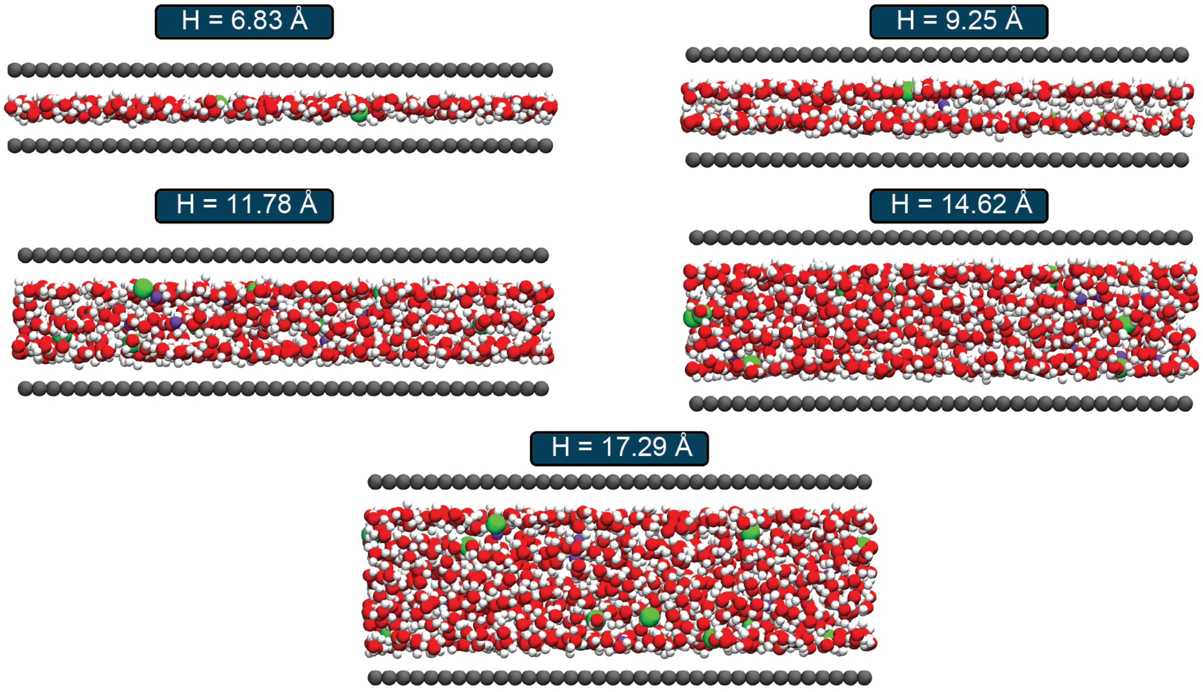

Figure S1: Snapshots of each slit height studied.

1.2 Machine learning potential validation

In this work, we use a Behler-Parrinello neural network potential (NNP) developed in our previous work[S1] to model sodium chloride solutions confined in graphene slit pores at the revPBE-D3 level of theory. This model has already been extensively validated based on energy/force errors relative to the underlying density functional theory as well as comparison to *ab initio* molecular dynamics (AIMD) trajectories. In addition to the benchmarking

provided in this past work, we provide a comparison of the velocity autocorrelation functions obtained from AIMD with those generated from our NNP in Fig. S2. The AIMD trajectories used for this comparison were generated in our previous work[S1] and consist of a 10 Å slit at 330K with 33 water molecules and 1 ion pair. The excellent agreement between our model and AIMD demonstrates that the NNP can accurately capture diffusion in these nanoconfined electrolytes.

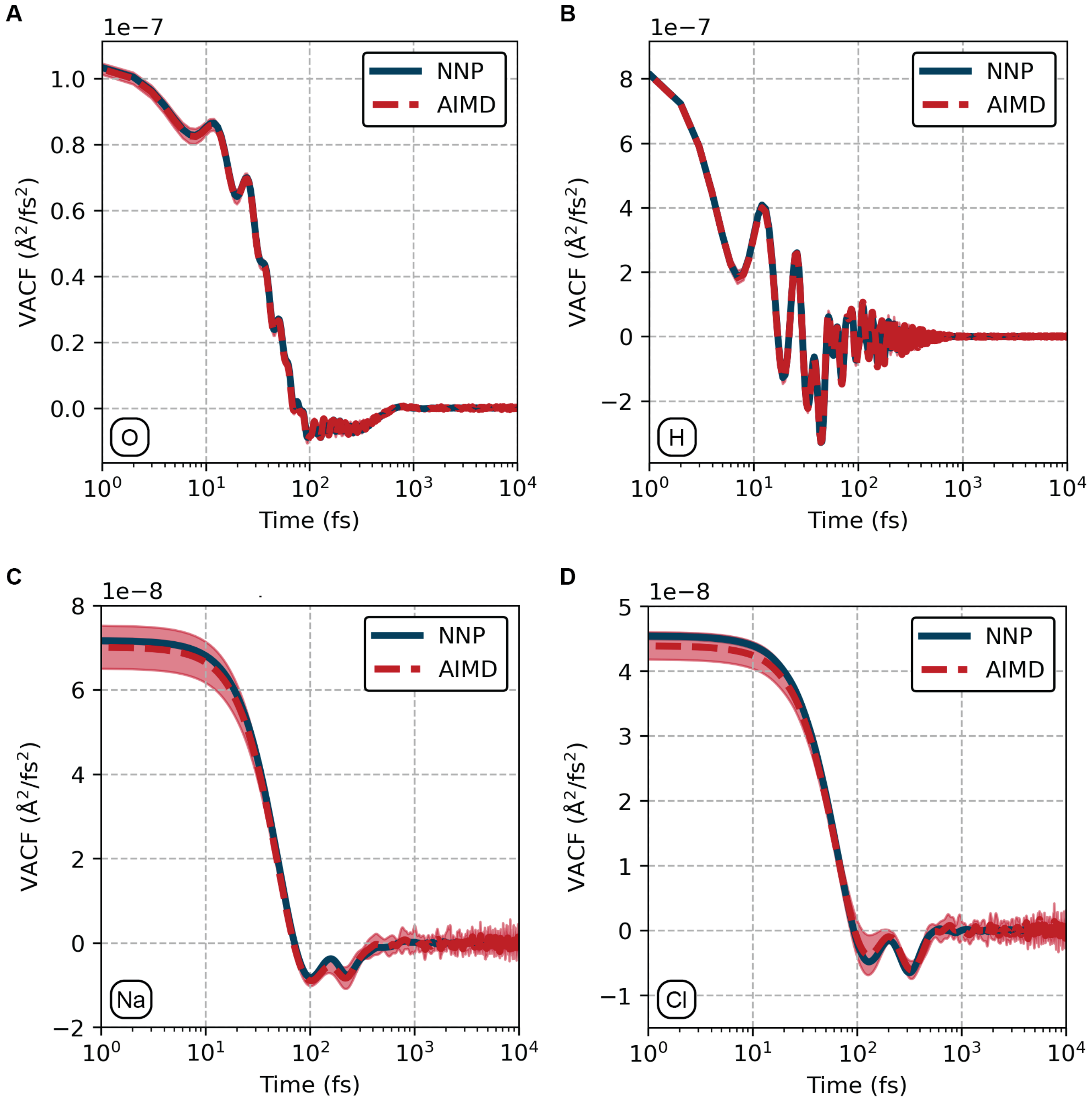

Figure S2: Comparison of velocity autocorrelation functions (VACFs) obtained from *ab initio* molecular dynamics (AIMD) and the neural network potential (NNP) used herein for (A) oxygen, (B) hydrogen, (C) sodium, and (D) chlorine atoms.

Note that our simulations are driven by a committee of eight independently-trained NNPs, and that the disagreement between the committee members can be used as a proxy for the model uncertainty.[S2] For all simulation trajectories analyzed in this work, we observe that the standard deviation among the committee members' energy predictions was less than 0.18 meV/atom. This low committee error demonstrates that the model is not extrapolating beyond the region of configuration space on which it was trained and is thus producing accurate predictions.

Finally, we further validate the model by comparing the bulk electrolyte conductivity predicted by our model to experimental data[S3] at 300K and a concentration of 1M. As shown in Fig. S3, we observe excellent agreement, suggesting that the revPBE-D3 functional used to train our NNP satisfactorily captures the delicate balance of water-water, ion-water, and ion-ion water interactions that gives rise to macroscopic transport properties.

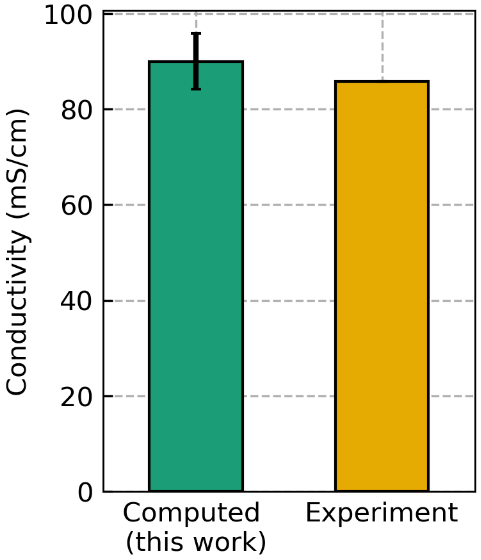

Figure S3: Conductivity of a 1M aqueous NaCl solution at 300K computed with the NNP used in this work and measured experimentally.[S3]

2 Additional system characterization

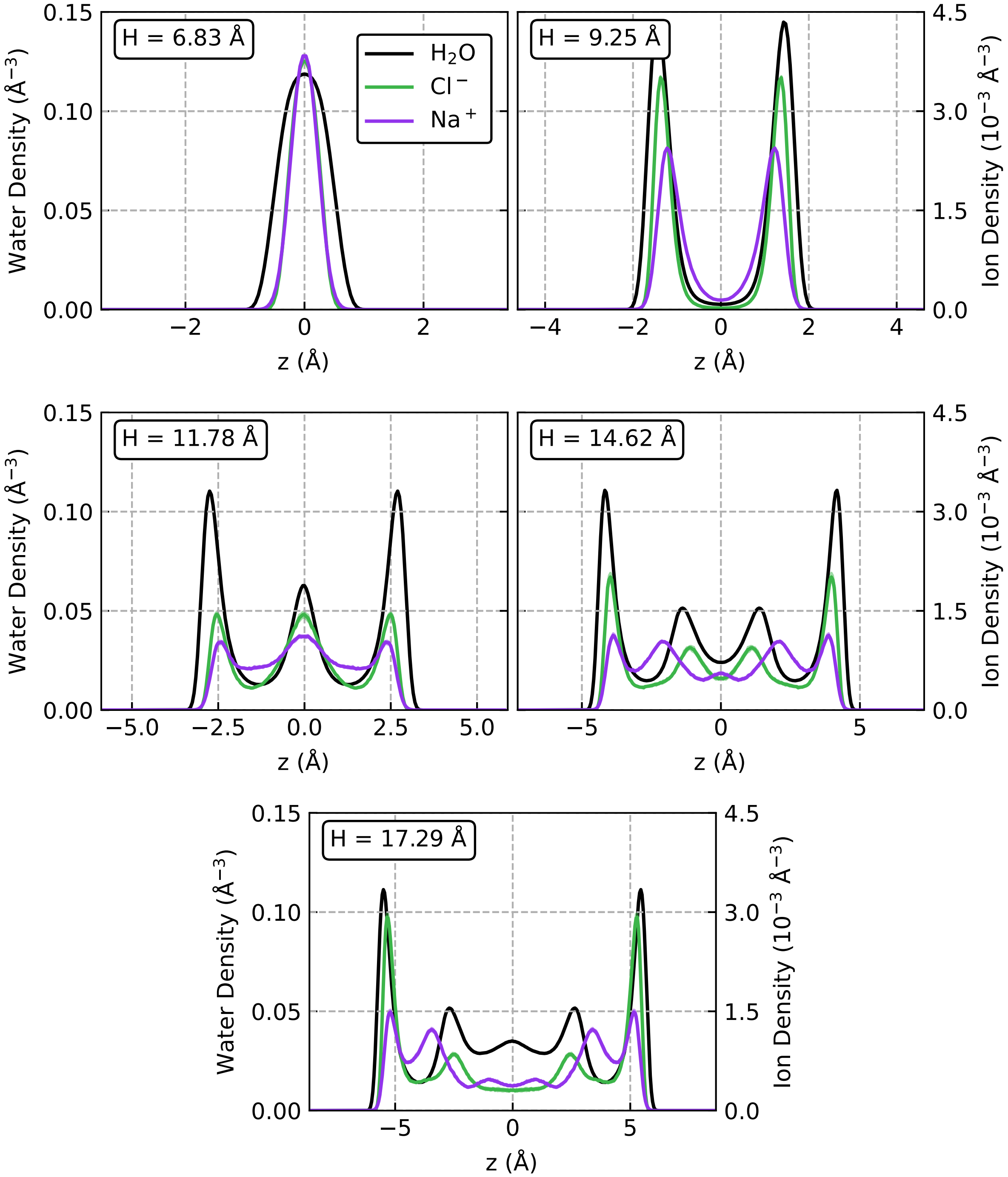

Figure S4: Density profiles along the height of each slit studied. All curves have been symmetrized around the center of the slit ($z = 0$), and the water curve is obtained from the density of oxygen atoms. The horizontal axis limits in each plot correspond to the position of the graphene layers.

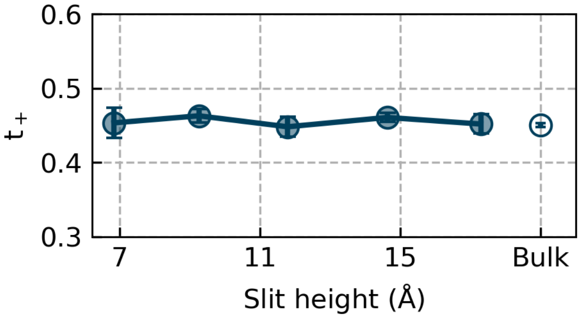

Figure S5: Cation transference number as a function of slit height.

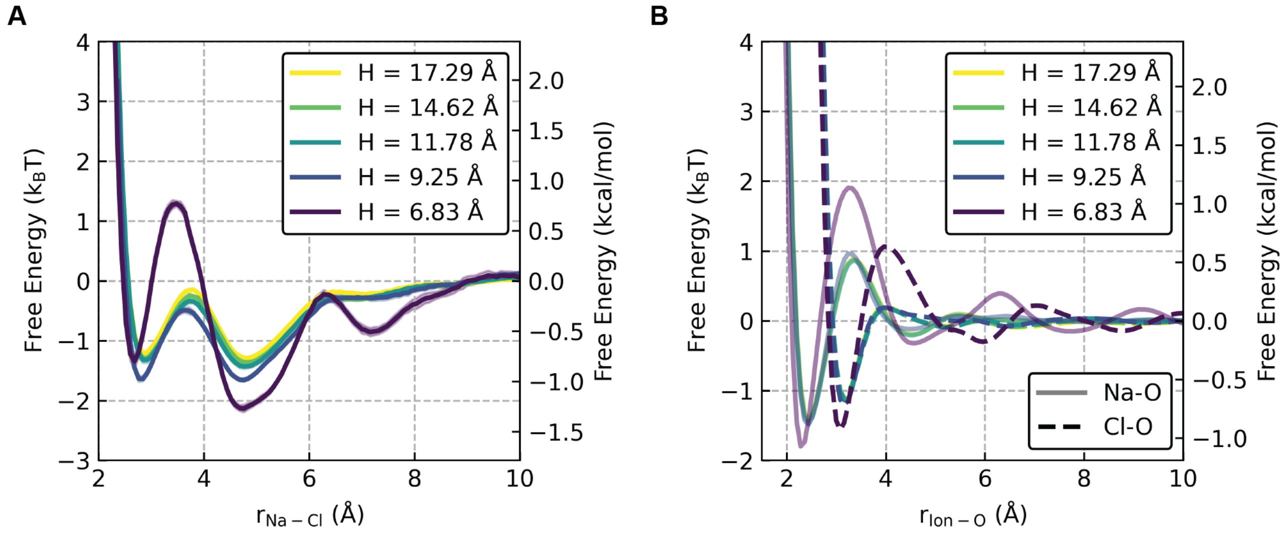

Figure S6: Potentials of mean force (PMFs) for (A) sodium and chloride ions and (B) ions and water molecules. PMFs were computed according to the approach given in Fong et al.[S1]

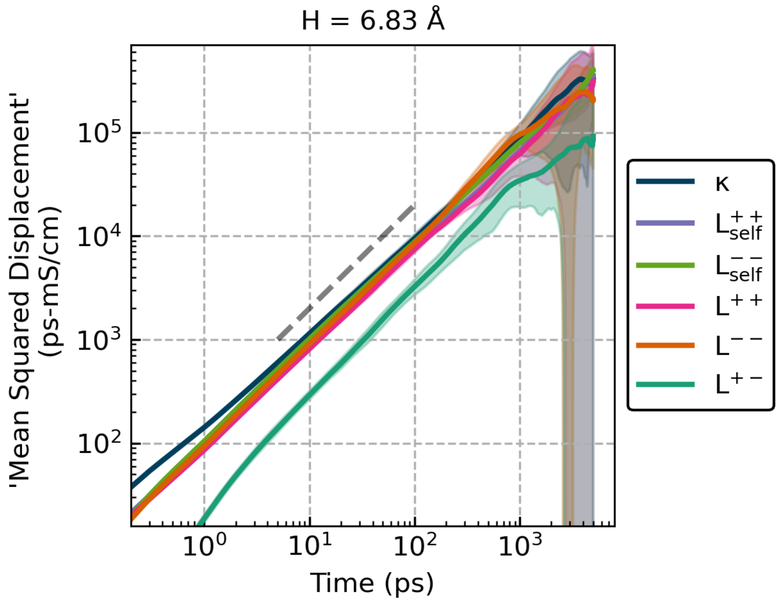

Figure S7: Representative curves for the quantities in angular brackets in Eqs. (3) and (4) for each transport coefficient (denoted "mean squared displacements"). Data is shown for the $H = 6.83$ Å system. The dashed line has a slope of one and is provided for reference, and the shaded regions correspond to the standard deviation among five independent replicate simulations.

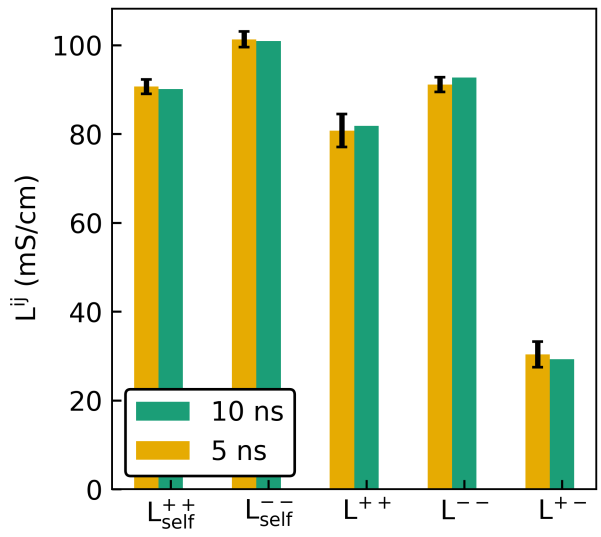

Figure S8: Comparison of each of the transport coefficients computed from the 5 ns runs used throughout this work with a 10 ns run for the $H = 6.83$ Å system. The agreement between the two sets of data confirms that our 5 ns simulations are long enough to extract converged results.

Table S2: Computed values of β upon fitting each of the transport coefficients, where β is the slope of the log-log plot of the "mean squared displacements" ($\left\langle \sum_\alpha [\boldsymbol{r}_i^\alpha(t) - \boldsymbol{r}_i^\alpha(0)] \cdot \sum_\beta [\boldsymbol{r}_j^\beta(t) - \boldsymbol{r}_j^\beta(0)] \right\rangle$ and $\sum_\alpha \left\langle [\boldsymbol{r}_i^\alpha(t) - \boldsymbol{r}_i^\alpha(0)]^2 \right\rangle$) over time, fitted over a time range of 1 ps to 100 ps. A β value of one corresponds to diffusive transport.

	Slit height (Å)					
	6.83	9.25	11.78	14.62	17.29	**Average**
L^{++}_{self}	0.94	0.97	0.97	0.98	0.97	**0.97**
L^{--}_{self}	0.93	0.96	0.97	0.97	0.98	**0.96**
L^{++}	0.96	0.93	0.96	0.95	0.96	**0.95**
L^{--}	0.97	0.99	1.00	0.98	0.96	**0.98**
L^{+-}	1.05	1.08	1.08	1.02	1.14	**1.07**

.

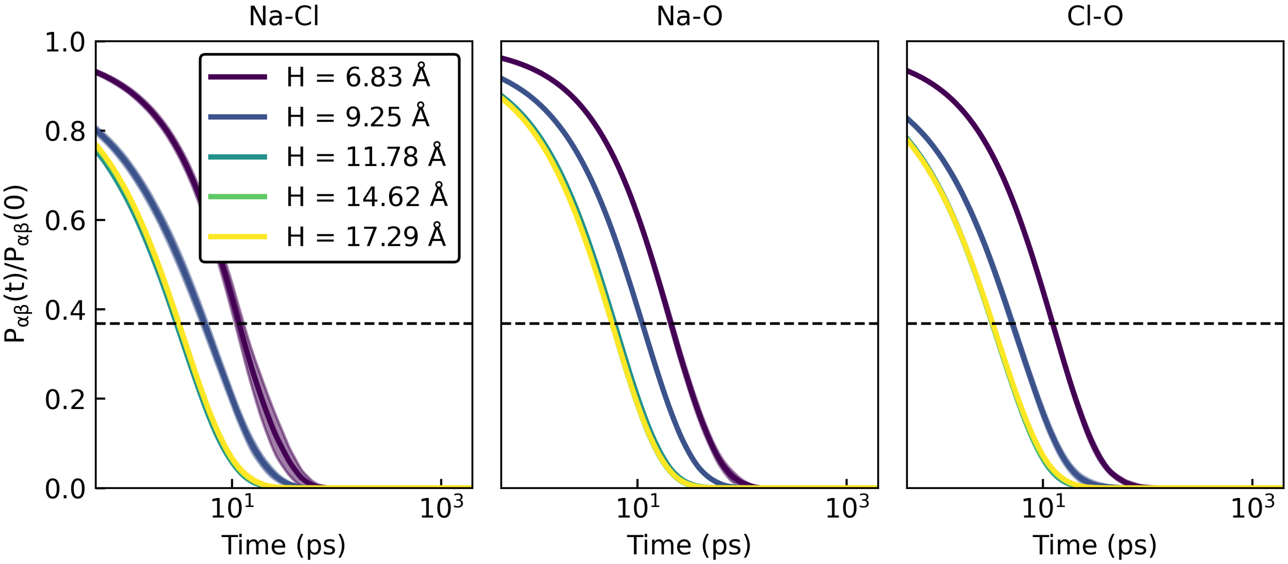

Figure S9: Normalized correlation functions $P_{\alpha\beta}(t) = \left\langle H_{\alpha\beta}(t)H_{\alpha\beta}(0)\right\rangle$ for each slit, where $H_{\alpha\beta}(t)$ is one if particles α and β are neighbors at time t and zero otherwise. The quantities $\alpha, \beta =$ Na, Cl (left panel), Na, O (middle panel), and Cl, O (right panel). Pair lifetimes are defined as the time for the correlation function to decay to a value of $1/e$, indicated by the dashed horizontal line in each panel.

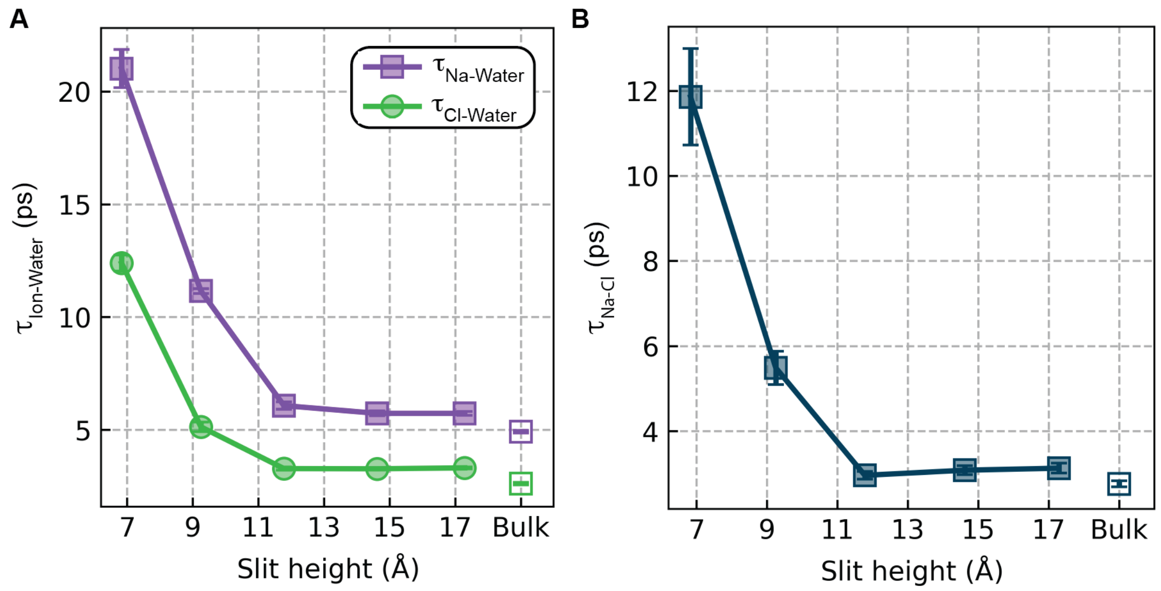

Figure S10: Pair lifetimes as a function of slit height extracted from the correlation functions in Fig. S9. (A) Ion-water residence times. (B) Na-Cl contact ion pair residence times.

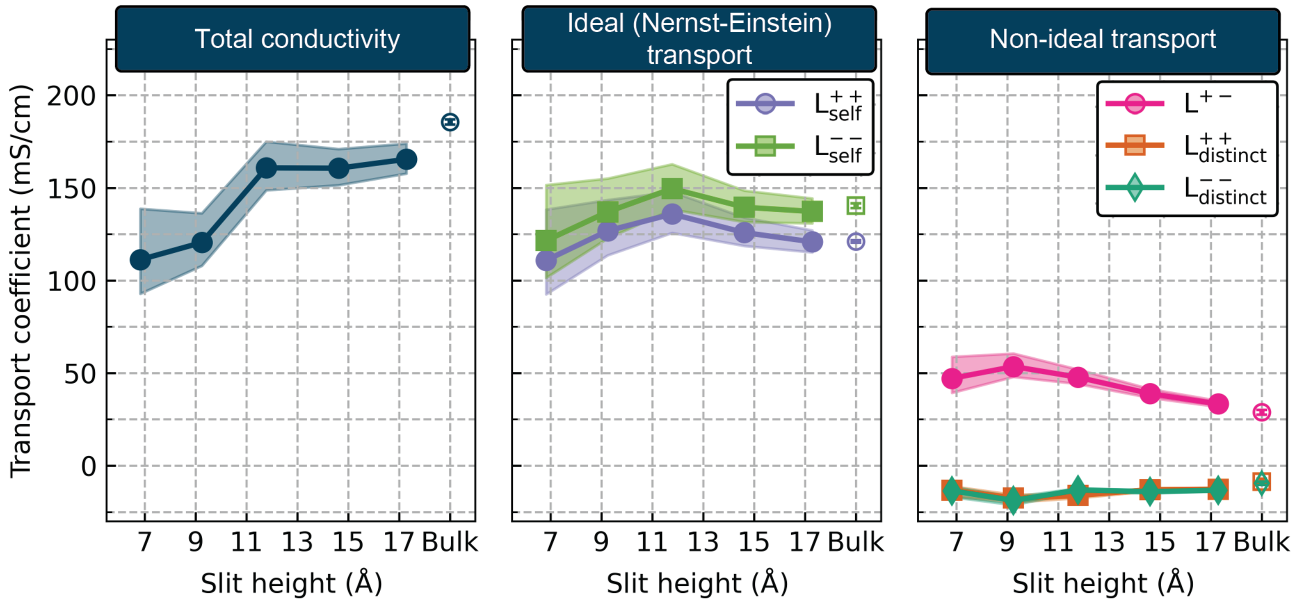

Figure S11: Impact of the choice of volume V on each of the transport coefficients. We define $V = L_x L_y (H - 2r_\text{C})$. The markers in each panel correspond to the data shown in the main text, where $r_\text{C} = 1.7$ Å, the experimental van der Waals radius of carbon. The limits of the shaded regions correspond the change in each transport coefficient upon changing r_C by $\pm 20\%$. We find that the transport trends are robust to these changes in V.

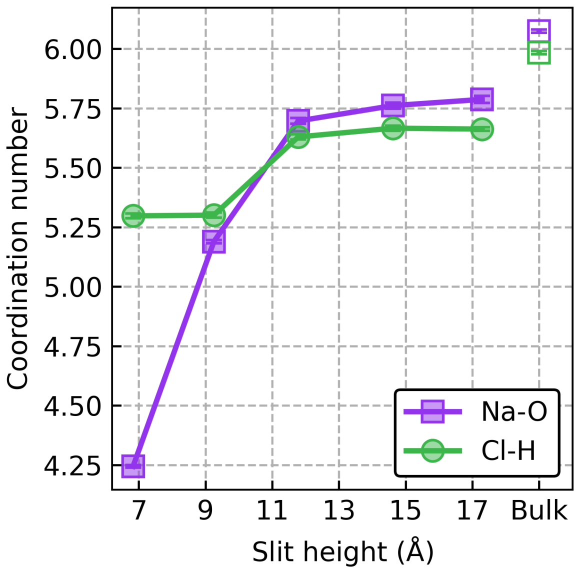

Figure S12: Ion coordination numbers as a function of slit height.

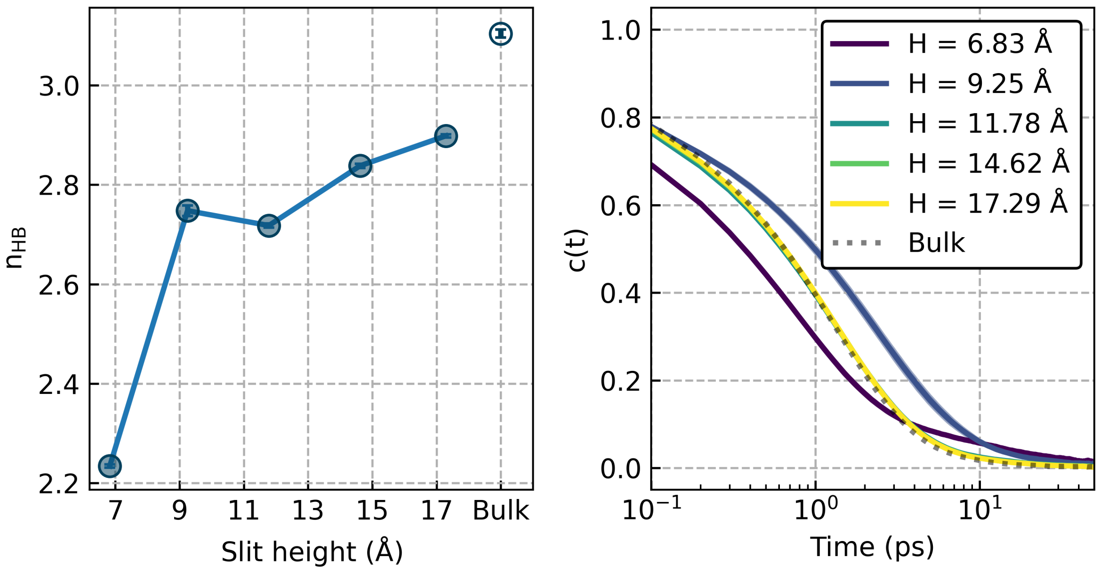

Figure S13: Water hydrogen bonding behavior. The left panel gives the number of water hydrogen bonds as a function of slit height. As the degree of confinement increases, we generally see a decrease in the number of hydrogen bonds between water molecules. We hypothesize that the slight deviation in this trend for the bilayer slit is related to the anomalous hydrogen-bonding configurations that have been noted previously in force field-based simulations of bilayer water.[S4] The right panel gives the hydrogen bond lifetime autocorrelation function $c(t) = \langle h(t)h(0)\rangle / \langle h \rangle$, where $h(t)$ is 1 if a given pair of molecules is hydrogen bonded at time t and 0 otherwise. The structural criteria for identifying hydrogen bonds and $c(t)$ are taken from Luzar and Chandler.[S5]

3 Finite size effects

In bulk fluids, finite size corrections to the self-diffusion coefficients are well-established:[S6] Yeh and Hummer[S7] derived an analytic expression based on hydrodynamics predicting that the finite size correction should scale as $1/L$, where L is the box length. Finite size effects for the Onsager transport coefficients L^{ij}, however, have not been well-explored. Of the limited work that has investigated finite size effects for L^{ij},[S8] we are aware of none that has developed either analytical or empirical scaling relations for the finite size correction as a function of L. Additional nuances arise when considering finite size effects in confined geometries. Simonnin et al.[S9] derived an analytical expression for the center-of-mass diffusion coefficient of a fluid confined to a slit pore in which the finite size correction scales as $1/L^2$. Note that this derivation, however, assumed a no-slip boundary condition, which is not appropriate for the water/graphene interfaces in this work. In contrast, Zaragoza et al.[S10] suggested that no finite size correction is necessary in confined systems if one considers diffusion relative to the center-of-mass motion of the fluid (as is done in this work).

Given the lack of consensus on finite size effects for L^{ij} and for confined fluids, herein we empirically investigate the transport properties of our nanoconfined electrolytes as a function of the size of the graphene sheets. As shown in Table S1, our graphene sheets are not perfectly square, so we take L to be the average of the x- and y- dimensions of the sheet. Note that the number of different L-values that we can simulate is limited by both the computational cost of the NNP-based simulations (which prevent us from going to very high L) as well as the need to keep the overall ion concentration constant (which limits the lowest value of L we can use). We investigate L-values of approximately 35 Å, 45 Å (the system size discussed in the main text), 70 Å, and 90 Å. For each of these L-values, we have generated five replicate simulations of at least 1.5 ns each.

Each of the transport coefficients is plotted as a function of $1/L$ in Fig. S14 for two representative slit heights (the largest and smallest slits studied in this work). We observe that for all slit heights, the self-diffusion coefficients of each species, as well as L^{++}, L^{+-}, and L^{--}, are found to change linearly with $1/L$, as in bulk systems. Furthermore, as in numerous prior works simulating bulk electrolytes,[S8,S11,S12] we find that the overall ionic conductivity is not systematically influenced by finite size effects (Fig. S15). This lack of finite size effects for the conductivity arises from the fact that the plots of L^{++}, L^{+-}, and L^{--} vs $1/L$ are all found to have essentially the same slope, such that by Eq. (1) the L-dependence cancels out.

In order to correct for these finite size effects, we extrapolate the plots of each transport coefficient vs $1/L$ to the limit of infinite L. When fitting this data via linear regression, the average R^2 across all slits and transport coefficients is 0.96 and the minimum value is 0.92. Note that the finite size correction is found to vary with slit height, such that explicit finite size tests must be done for each degree of confinement. In the main text, all transport coefficients and diffusion coefficients have been corrected for finite size effects in this manner. Other properties, such as coordination numbers and residence times, are computed based on the L =45 Å system, as this is the largest system size for which it is computationally feasible to perform converged, long-time simulations (five replicates of five nanoseconds each).

Fig. S16 compares the transport coefficient data with and without finite size effect corrections, demonstrating that finite size corrections only introduce minor changes to the trends in transport.

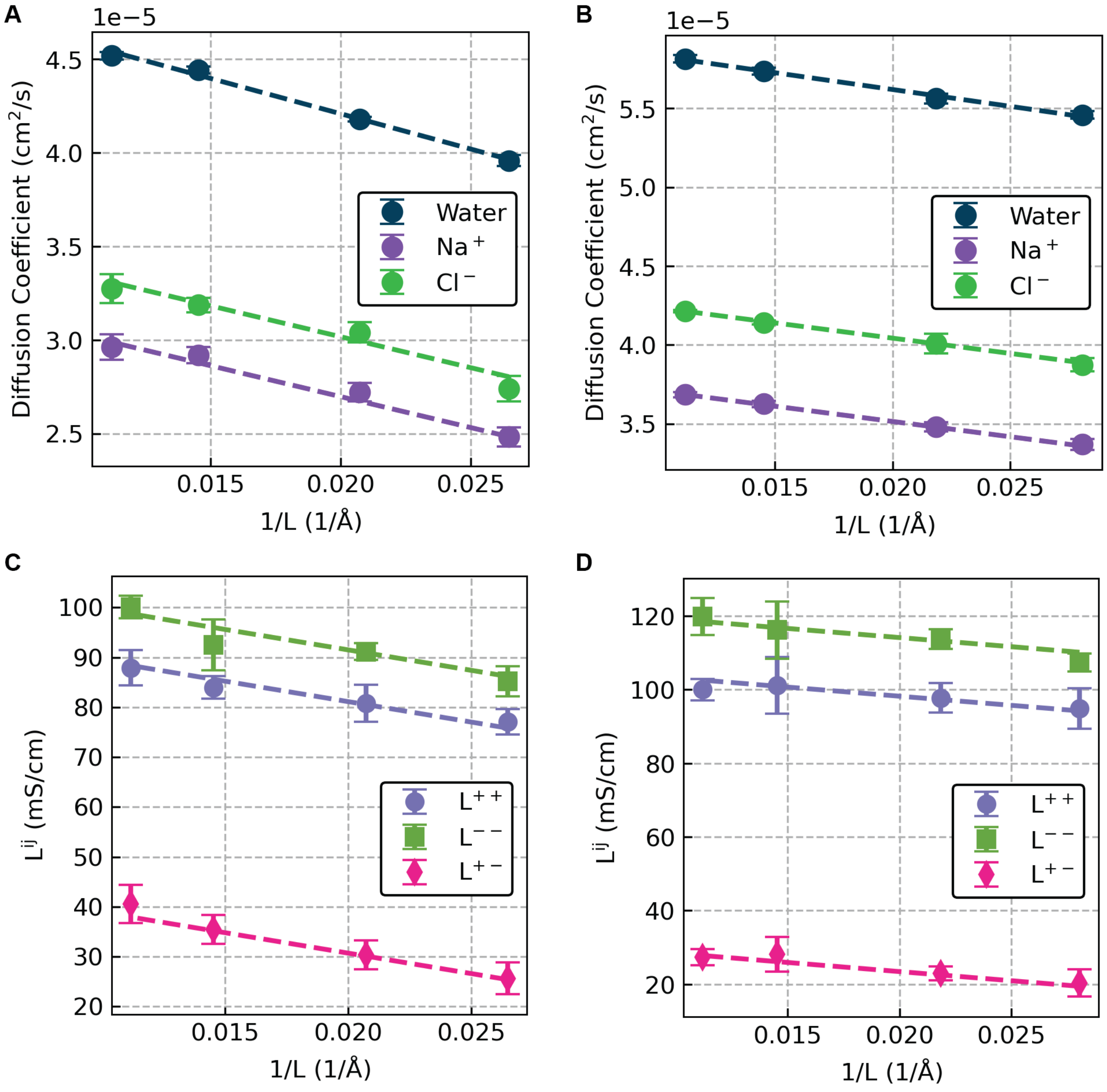

Figure S14: Diffusion coefficients (A, B) and Onsager transport coefficients (C, D) as a function of $1/L$, where L is the average of the x- and y- dimensions of the graphene sheet. Panels (A) and (C) correspond to the $H = 6.83$ Å system, and panels (B) and (D) correspond to the $H = 17.29$ Å system.

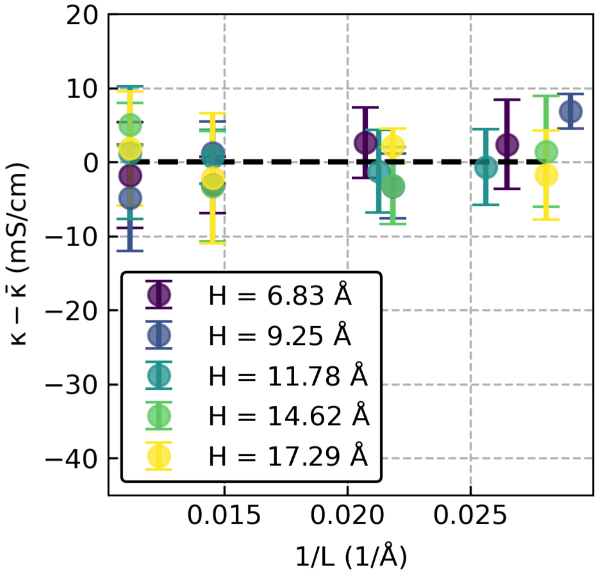

Figure S15: Conductivity of each slit height as a function of $1/L$, where L is the average of the x- and y- dimensions of the graphene sheet. For each slit height, we shift the data relative to the average conductivity across all L-values. No systematic changes in conductivity are observed with respect to box size.

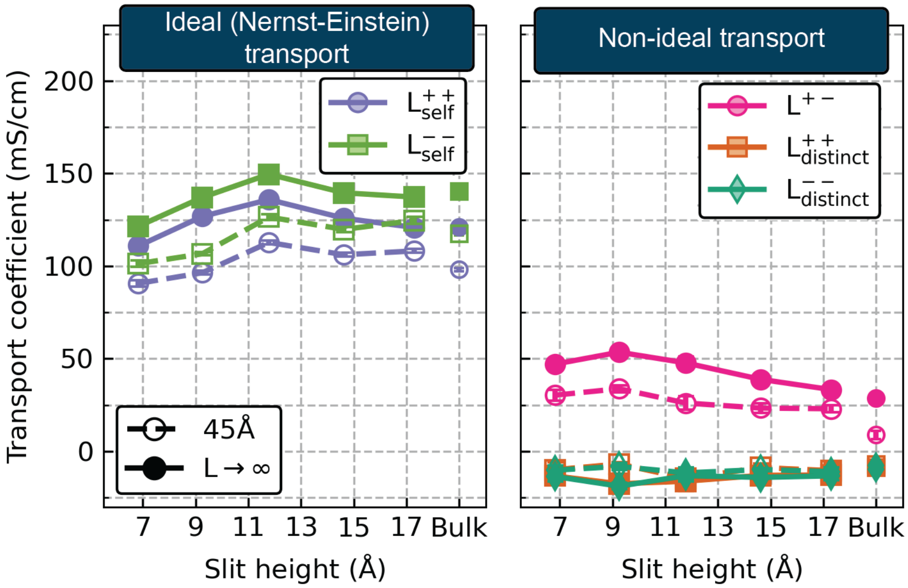

Figure S16: Transport coefficients with (solid markers) and without (unfilled markers) finite size correction. The data without a finite size correction corresponds to the $L = 45$ Å system. Note that the total conductivity is not subject to finite size effects.

4 Fixed density simulations

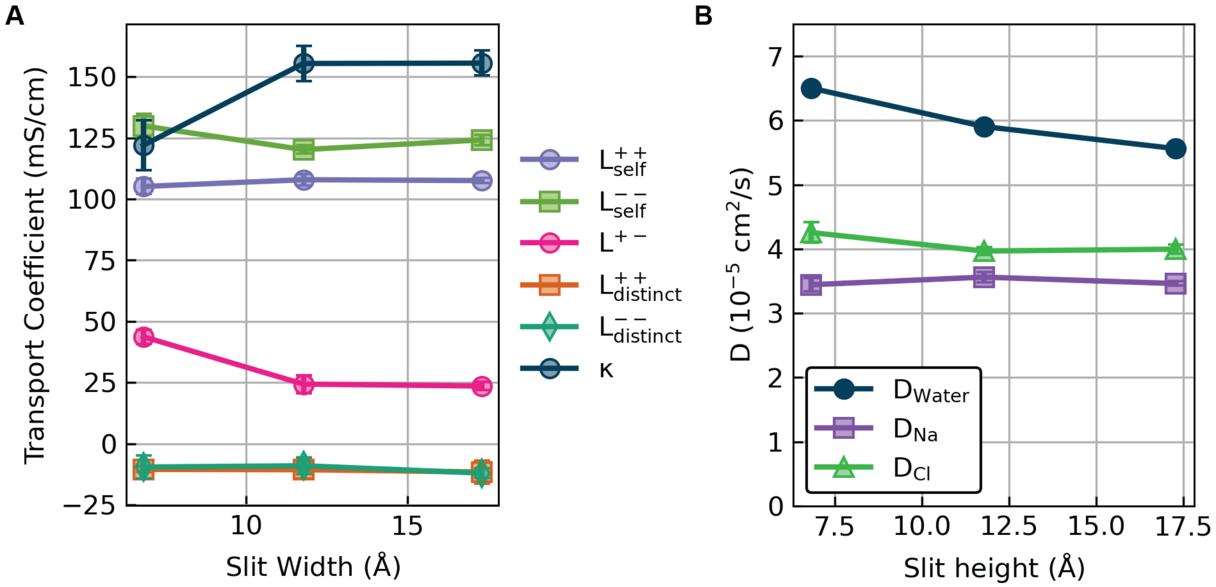

Figure S17: Transport behavior of slits in which the total electrolyte density has been fixed to a constant value of 1.01 g/cm^3 (the equilibrium density for the largest slit studied in this work, H = 17.29 Å). (A) Total conductivity and Onsager transport coefficients. (B) Self-diffusion coefficients. Note that this data has not been corrected for finite-size effects.

Fig. S17 summarizes the transport behavior of electrolytes with a fixed density, rather than the equilibrium density obtained from the piston-based equilibration method described in the Methods section. The density of each system is 1.01 g/cm^3, which corresponds to the equilibrium density for the H = 17.29 Å slit. As in the main text, the density is defined based on the following definition of the electrolyte volume: $V = L_x L_y (H - 2r_C)$, where r_C = 1.7 Å, the experimental van der Waals radius of carbon. The system sizes and number of molecules in each system are reported in Table S3.

Table S3: Slit dimensions and number of water molecules and ion pairs for simulations with the density fixed at 1.01 g/cm^3.

Slit Height (Å)	Graphene dimensions (Å x Å)	Number of waters	Number of NaCl
6.83	49.40 x 51.34	277	5
11.78	44.46 x 47.06	559	10
17.29	44.46 x 47.06	926	17

We observe that the choice of electrolyte density does not qualitatively influence the overall conductivity trend, or the trends in L^{+-}, L^{++}_{distinct}, and L^{--}_{distinct}. However, the fixed density simulations yield relatively constant trends in L^{++}_{self} and L^{--}_{self}, reinforcing our conclusion from Fig. 2 that density is the main factor influencing ion diffusion. We additionally observe that the water self-diffusion coefficients in these simulations increase as the degree of confinement

increases. We hypothesize that this trend is related to the results shown in Fig. 4B, in which interfacial water diffuses faster than water in the center of the slit. As the slits become more confined, the fraction of water molecules at the interface increases, thereby increasing the average self-diffusion coefficient.

5 Transport at 300K

As described in the Methods section, all data reported in the main text were obtained from simulations performed at 350K. This choice of temperature was informed by the complex phase behavior of the bilayer electrolyte observed at 300K. As illustrated in Fig. S18A, we see ice-like regions with hexagonal and pentagonal rings of water molecules coexisting with liquid-like regions containing the NaCl ions. These regions are dynamic, rearranging over the course of several hundred picoseconds. This behavior is in line with previous work reporting the high melting temperature of bilayer ice[S4] and results in anomalously slow diffusion in the electrolyte, as shown in Fig. S18B. While the interesting behavior of the bilayer electrolytes at 300K certainly warrants further study, in this work we chose to use a temperature of 350K to systematically compare transport as a function of slit height without the convoluting influence of phase transitions.

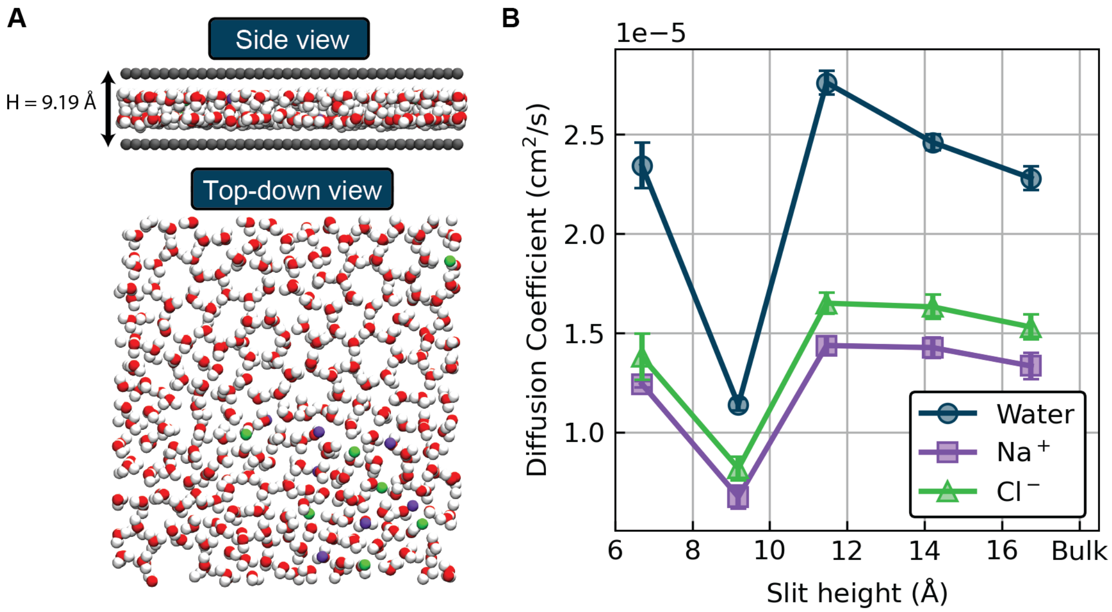

Figure S18: Behavior of the bilayer electrolyte at 300K. (A) Snapshot of the ice-like regions formed in the bilayer system (upper right region of the image). (B) Anomalously slow diffusion in the bilayer electrolyte, where $H = 9.2$ Å. Note that this data has not been corrected for finie-size effects.

References

(S1) Fong, K. D.; Sumic, B.; O'Neill, N.; Schran, C.; Grey, C. P.; Michaelides, A. The interplay of solvation and polarization effects on ion pairing in nanoconfined electrolytes. *Nano Letters* **2024**, *24*, 5024–5030.

(S2) Imbalzano, G.; Zhuang, Y.; Kapil, V.; Rossi, K.; Engel, E. A.; Grasselli, F.; Ceriotti, M. Uncertainty estimation for molecular dynamics and sampling. *The Journal of Chemical Physics* **2021**, *154*.

(S3) Miller, D. G. Application of irreversible thermodynamics to electrolyte solutions. i. determination of ionic transport coefficients lij for isothermal vector transport processes in binary electrolyte systems1, 2. *The Journal of Physical Chemistry* **1966**, *70*, 2639–2659.

(S4) Kastelowitz, N.; Johnston, J. C.; Molinero, V. The anomalously high melting temperature of bilayer ice. *The Journal of chemical physics* **2010**, *132*.

(S5) Luzar, A.; Chandler, D. Hydrogen-bond kinetics in liquid water. *Nature* **1996**, *379*, 55–57.

(S6) Celebi, A. T.; Jamali, S. H.; Bardow, A.; Vlugt, T. J.; Moultos, O. A. Finite-size effects of diffusion coefficients computed from molecular dynamics: a review of what we have learned so far. *Molecular Simulation* **2021**, *47*, 831–845.

(S7) Yeh, I.-C.; Hummer, G. System-size dependence of diffusion coefficients and viscosities from molecular dynamics simulations with periodic boundary conditions. *The Journal of Physical Chemistry B* **2004**, *108*, 15873–15879.

(S8) Shao, Y.; Shigenobu, K.; Watanabe, M.; Zhang, C. Role of viscosity in deviations from the nernst–einstein relation. *The Journal of Physical Chemistry B* **2020**, *124*, 4774–4780.

(S9) Simonnin, P.; Noetinger, B.; Nieto-Draghi, C.; Marry, V.; Rotenberg, B. Diffusion under confinement: Hydrodynamic finite-size effects in simulation. *Journal of Chemical Theory and Computation* **2017**, *13*, 2881–2889.

(S10) Zaragoza, A.; González, M. A.; Joly, L.; López-Montero, I.; Canales, M.; Benavides, A.; Valeriani, C. Molecular dynamics study of nanoconfined TIP4P/2005 water: how confinement and temperature affect diffusion and viscosity. *Physical Chemistry Chemical Physics* **2019**, *21*, 13653–13667.

(S11) Gullbrekken, Ø.; Røe, I. T.; Selbach, S. M.; Schnell, S. K. Charge transport in water–NaCl electrolytes with molecular dynamics simulations. *The Journal of Physical Chemistry B* **2023**, *127*, 2729–2738.

(S12) Blazquez, S.; Abascal, J. L.; Lagerweij, J.; Habibi, P.; Dey, P.; Vlugt, T. J.; Moultos, O. A.; Vega, C. Computation of electrical conductivities of aqueous electrolyte solutions: Two surfaces, one property. *Journal of chemical theory and computation* **2023**, *19*, 5380–5393.
